# Fruit‐based drink sensory, physicochemical, and antioxidant properties in the Amazon region: Murici (*Byrsonima crassifolia *(L.) *Kunth* and *verbascifolia *(L.) DC) and tapereba (*Spondia mombin*)

**DOI:** 10.1002/fsn3.1520

**Published:** 2020-04-15

**Authors:** Vanessa Rosse de Souza, Adriana Aniceto, Joel Pimentel Abreu, Julia Montenegro, Bruno Boquimpani, Vanessa Azevedo de Jesuz, Monique de Barros Elias Campos, Paulo Sérgio Marcellini, Otniel Freitas‐Silva, Rafael Cadena, Anderson Junger Teodoro

**Affiliations:** ^1^ Laboratory of Functional Foods Universidade Federal do Estado do Rio de Janeiro Rio de Janeiro Brazil; ^2^ Departamento de Bioquímica Instituto Biomédico Universidade Federal do Estado do Rio de Janeiro Rio de Janeiro Brazil; ^3^ Embrapa Agroindústria de Alimentos Empresa Brasileira de Pesquisa Agropecuária Rio de Janeiro Brazil; ^4^ Laboratory of Sensory Analisis Universidade Federal do Estado do Rio de Janeiro Rio de Janeiro Brazil

**Keywords:** Amazon biome, bioactive compounds, exotic fruits, functional foods

## Abstract

Increased fruit consumption due its protective effect on the organism is accompanied by the development of the processing industry of these products. The aim of this work was to optimize fruit pulp‐based beverage formulations from the murici and tapereba Amazon region, taking into account their sensory acceptance and antioxidant activity. Total soluble solid content, reducing sugar content, titratable acidity contents, pH, and ascorbic acid content were determined in pulps and formulations. The total content phenolic compounds and antioxidant activity were also evaluated. A 2^2^ factorial experiment was formulated to optimize ingredients for the production of murici and tapereba fruit drinks. The murici pulp had higher acidity and higher ascorbic acid content. The analysis of phenolic compounds and antioxidant activity presented higher quantity in tapereba pulp. Tapereba‐based beverages had better acceptance by the evaluated criteria. Fruit‐based beverages murici and tapereba are a well‐accepted product and have important nutritional characteristics.

## Graphical abstract

Antioxidant activity high in fruit pulp murici and tapereba may be associated with their vitamin C and phenolic compounds content, as well as the presence of carotenoids in their composition. Murici and tapereba beverages are a well‐accepted product with significant nutritional features.

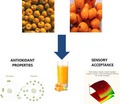

## 1 INTRODUCTION

Brazil produces many fruits consumed and appreciated worldwide. It also has a range of exotic and small known fruits belonging to the Amazonian biodiversity, which represents a great potential for the development of new products. Consumers around the world are very concerned about healthy eating habits, so fruit is associated with a primary source of nutrients and functional compounds (Tiburski et al., 2011). Tropical fruit consumption is increasing on the local and international market, providing producers with the opportunity to access places where consumers emphasize the exotic character and the presence of potential nutrients capable of preventing certain diseases (Rufino et al., 2010).

Murici and tapereba fruits are native to the Amazon region, and the fruits are mainly produced in natural forms, with good acceptance throughout the Amazon region and still little distribution in the country and on the international market (Lamarão, Leão, Soares, & Pieri, 2017).

In recent years, processed fruit and vegetable products’ consumption has increased. The increase is suggested by the time limitation of consumers and the practicality offered by this type of product (Leandro et al., 2011). The processing of pulp and fruit juices is an important agro‐industry activity to be insufflated as it adds economic value to the fruit, avoiding waste and minimizing losses that may occur during the marketing of the product in nature, in addition to being an alternative to the fruit producer. The growth of industrial production is mainly occurring due to the emergence of products that have the juice as a secondary ingredient, ice cream, and yogurt (Virgolin, Rosan, Seixas, & Janzantti, 2017).

Optimization of fruit concentrations in various formulations using the fruits murici and tapereba can result in a formulation with significant antioxidant capacity, giving the consumer benefits in addition to increased interest in the consumption of these products.

The aim of this study is to optimize drink formulations based on fruit pulps from the Amazon region murici and tapereba, taking into account their sensory acceptance and antioxidant activity, which contribute to the consumer's health.

## 2 MATERIALS AND METHODS

### 2.1 Sample

Pulp of murici and tapereba conditioned in plastic bags (1 kg), sealed, duly identified, and stored at controlled temperature (−18°C) were supplied by pulp processor from Pará (Brazil). The frozen pulp was transported under freezing conditions, obtained from the use of dry ice and portable thermal container until the Laboratory Analysis of Functional Foods (LAAF‐UNIRIO), Rio de Janeiro (Brazil), where they remained under frozen conditions (−18°C) until the moment of the analyses.

### 2.2 Physicochemical characterization

Proximate physicochemical composition of pulps and formulations of fruit‐based beverages murici and tapereba had the following analyses in triplicate: total titratable acidity, reducing sugars, total soluble solids (°Brix), pH, and vitamin C according to the official method (Association of Official Analytical Chemists 2000).

### 2.3 Color analysis

Color analysis in the pulps murici and tapereba was performed using a Konica Minolta CM‐5 digital colorimeter, with using the parameters L* (lightness), a* (red/green intensity), and b* (yellow/blue intensity) of the CIE Laboratory system (Commission Internationale de l’Eclairage). All analyses were performed in triplicate. The equipment was calibrated according to the manufacturer's instructions. Approximately 50 g of previously homogenized sample was used (Lima et al., 2015).

### 2.4 Total phenolic content

Total phenolic content was determined by the Folin–Ciocalteu method, which was adapted from Bernards et al. (2018). The drinks based on murici and tapereba and 2.5 ml Ciocalteu reagent solution 10% were combined and then mixed well using a vortex. The mixture was allowed to react for 5 min, then 2 ml of 4% sodium carbonate solution was added and mixed well, and the solution was incubated at room temperature (25°C) in the dark for 2 hr. The absorbance was measured at 750 nm using a spectrophotometer (Turner 340), and the results were expressed in gallic acid equivalents, mg/100 g organic grape peel flour using a gallic acid (2.5–50 µg/µl) standard curve.

### 2.5 DPPH assay

DPPH methanolic solution (0.06 mM) was mixed with 0.5 ml of pulps and formulations of fruit‐based beverages on murici and tapereba in the three concentrations. The reaction mixture was turned completely and left in the dark at controlled temperature for 60 min. The absorbance of the blend assays was measured applying a spectrophotometer (Turner 340) at 515 nm in triplicate (Abreu et al., 2019).

### 2.6 TEAC assay

TEAC assay was performed following the procedure proposed by Rufino et al. (2010). The ABTS radical (7 mM) was prepared and kept in the dark at room temperature for 16 hr before use. The ABTS solution was diluted with ethanol with an absorbance of 0.70 ± 0.02 at 734 nm. After adding 30 μl of pulps and formulations of fruit‐based beverages on murici and tapereba or Trolox standard to 3 ml diluted ABTS solution, the absorbance was recorded at six minutes after addition. The analyses were performed in triplicate on the spectrophotometer (Turner 340). The blank assay used ethanol and antiradical activity was expressed as μmol TE/g.

### 2.7 ORAC assay

ORAC assay was performed with samples of pulps and formulations of fruit‐based beverages on murici and tapereba, according to Prior and Cao, (2000). Buffered saline solution (PBS‐pH 7.4), fluorescein solution, Trolox standard, and 2,2'‐azobis (2‐amidinopropane) dihydrochloride (AAPH) solution were prepared for this purpose. The Trolox standard was prepared at eight concentrations. For white aliquots and control, the PBS solution was used. The Trolox standard and fruit‐based beverages murici and tapereba were added to the plate in increasing concentration and in duplicate. Then, 120 μl of the fluorescein solution was added, and then, the AAPH solution was added to all wells, except for the control. The fluorescence drop reading was measured using an automated plate reader (SpectraMax i3x, Molecular Devices) with 96‐well plates at 485/520 nm.

### 2.8 Experimental design

The production of the formulations for the drinks was carried out according to the proportions for each independent variable (Table S1). In order to optimize the developed murici‐ and tapereba‐based drinks, factorial design 2^2^ was used, in which the independent variables were concentration of tapereba or murici pulps in percentage (X^1^) and sugar concentration in percentage (X^2^) and the dependent variables were the appearance (Y^1^), flavor (Y^2^), texture (Y^3^), and overall impression (Y^4^). A total of eleven formulations were produced, containing four axial points and three central points, producing a total of eleven samples for use in the acceptance tests (Rodrigues & Iemma, 2005).

### 2.9 Sensory evaluation

Sensorial evaluation of drinks based on murici and tapereba was carried out by 85 consumers. The parameters included in the analysis were appearance, taste, texture, and overall impression. The methodology used a hedonic scale of nine points ranging from 1 (not very liking) to 9 (really enjoyed) (Abreu et al., 2019). Sensory analyses were performed in the Laboratory of Sensory Analysis of Federal University of the State of Rio de Janeiro (LASEN). The data were collected by means of paper sheets. Sensory panelists of consumers were recruited for the evaluation. The tasters were recruited for their availability, interest, and frequency of consumption. The majority of the panel was composed of faculty, staff, and students from the Federal University of the State of Rio de Janeiro. It received approximately 30ml of each product sample in disposable plastic cups encoded with three‐digit temperature‐controlled random numbers in monadic form and using a complete balanced block design (Macfie, Bratchell, Greenhoff, & Vallis, 1989). The research protocol of the study was approved by the Research Ethics Committee of the University of the State of Rio de Janeiro (CAAE no. 39693914.8.0000.5285).

### 2.10 Statistical analysis

The univariate analysis of variance (ANOVA) with the Tukey post‐test at a 95% confidence for antioxidant assays and Dunn's for sensory tests were used to determine and compare the statistical differences, depending on the distribution of data. Experimental data were analyzed statistically using STATISTICA 7.0 (StatSoft, South America). The results were expressed as the mean and standard deviation.

## 3 RESULTS AND DISCUSSION

### 3.1 Physicochemical analysis

The pH and titratable acidity results for tapereba pulp were, respectively, 2.60 ± 0.01 and 1.74 ± 0.05 g/100 g. Murici pulp showed lower acidity than tapereba pulp, with titratable acidity equivalent to 0.75 ± 0.03 g/100 g and pH equivalent to 3.36 ± 0.01 (*p* < .05) (Table 1).

**Table 1:** Physicochemical characterization of in nature pulps of murici and tapereba

Parameter	Murici	Tapereba
Acidity (g/100 g)	0.75 ± 0.03^a^	1.74 ± 0.05^b^
Reducing Sugar (g/100 g)	3.79 ± 0.14^a^	9.90 ± 0.43^b^
Vitamin C (mg/100 g)	58.88 ± 1.63^a^	25.93 ± 1.65^b^
Soluble Solids (°Brix)	4.20 ± 0.01^a^	9.80 ± 0.10^b^
pH	3.36 ± 0,01^a^	2.60 ± 0.01^b^
L* (lightness)	18.40 ± 0.03^a^	6.41 ± 0.03^b^
a* (a axis)	13.54 ± 0.02^a^	7.81 ± 0.04^b^
b* (b axis)	12.17 ± 0.02^a^	4.11 ± 0.01^b^
C* (chroma)	165.68 ± 0.01^a^	38.93 ± 0.33^b^
h* (matrix angle)	0.90 ± 0.31^a^	0.53 ± 0.01^b^

Note

Results expressed as mean ± standard deviation. Different letters on the same line indicate significant difference (*p* < .05).

Acidity is one of the criteria affecting the flavor‐based classification of fruits; fruits with citric acid levels ranging from 0.08% to 1.95% can be classified as light‐flavored and are well accepted for fresh fruit consumption (Souza, Aparecida, Pereira, Queiroz, & Borges, 2012). Despite having acidic characteristics, murici and tapereba pulps fall into this evaluation category.

Livre et al. (2010) evaluated nature pulp of fruits of the Amazon and found values of pH (3.7 ± 0.00; 2.9 ± 0.4) and total acidity (1.0 ± 0.1; 1.3 ± 0.1%) for murici and tapereba similar to those found in the present study. Tiburski et al. (2011) found values of pH (2.83 ± 0.01), titratable acidity (1.46 ± 0.01 g/100 g) and soluble solids (10.2 ± 0.1 °Brix) like to those found in our study for tapeworm pulp.

Pulp of tapereba presented a higher content of reducing sugars (9.90 ± 0.43 g/100 g) and soluble solids (9.80 ± 0.10 °Brix) when compared to the murici pulp (4.20 ± 0.01 g/100 g and 3.79 ± 0.14 °Brix, respectively) (*p* < .05) (Table 1).

The results of pH, titratable acidity, soluble solids, and vitamin C contents are observed within the levels established by means of murici and tapereba pulps’ identity and quality standards. The total soluble content of solids is associated with sugars and organic acids, a feature of interest as the consumer market prefers sweet fruit (Beckles, 2012).

A similar study found murici and tapereba pasteurized pulp values and found 1.5 ± 0.1 and 6.0 ± 0.7 °Brix values, respectively (Livre et al., 2010), and these values are lower than those found in the present study and may be related to the process of fruit maturation, according to da Silva et al. (2016). The increase of the soluble solids can be explained by the evaporation of water during the pasteurization process (Sales & Waughon, 2013).

Morzelle, Bachiega, Souza, Vilas Boas, and L.M.L. (2015), which analyzed Brazilian Cerrado fruits, including murici, was found to have a pH value of 4.74 ± 0.02, an acid value of 0.17 ± 0.01 g/100 g, and a sugar reducer of 2.97 ± 0.09g/100 g for the murici fruit.

Murici pulp was higher with vitamin C content (58.88 mg/100 g) compared to tapereba pulp (25.93 mg/100 g) (*p* < .05) (Table 1). This vitamin has an important antioxidant, derived primarily from fruits, and protects the body (Putchala, Ramani, Sherlin, Premkumar, & Natesan, 2013). Matietto, Lopes, and Menezes (2010) found vitamin C values for tapereba pulp from the same region of Brazil (23.72 ± 0.08 mg/100 g) similar to that found in the present study. Leandro et al. (2011) also obtained a similar result to vitamin C content for the murici pulp from Uberlandia/Brazil (47.44 ± 3.26 mg/100 g). Da Silva et al. (2016) observed in his analysis of murici pulp that the fruit maturity rate is directly related to its acidity and that the murici fruit can be considered an acidic fruit.

Colorimetric analysis showed that the murici pulp presented a more yellowish coloration when compared to the tapereba pulp (b* = 12.17 ± 0.02 and b* = 4.11 ± 0.01), varying the tonality to orange (h* = 0.90 ± 0.31 and h*= 0.53 ± 0.01). In relation to Chroma (C*), the murici pulp presented more vivid coloration (C* = 165.68 ± 0.01) and the pulp of tapereba more opaque staining (C* = 38.93 ± 0.33). The murici pulp had a lighter coloration (L* = 18.40 ± 0.03) and the darker taperebá pulp (L* = 6.41 ± 0.03) (*p* < .05) (Table 2).

**Table 2:** Antioxidant potential of murici and tapereba pulps evaluated by different assays

	Murici	Tapereba
DPPH (%)	50.77 ± 1.04^a^	55.78 ± 1.65^b^
TEAC (µM Trolox/g)	55.59 ± 2.21^a^	64.66 ± 2.76^b^
ORAC (mmol Trolox/g)	332.42 ± 86.81^a^	312.54 ± 88.95^a^
FRAP (µmol ferrous sulfate/g)	5.07 ± 0.01^a^	9.45 ± 0.76^b^

Note

Results expressed as mean ± standard deviation. Different letters on the same line indicate significant difference (*p* < .05).

Colorimetric evaluation is an important criterion for the physical evaluation of fruits, influenced by the processing and storage in which they are submitted. Therefore, its composition is affected by this parameter, for example, the chromaticity coordinate b *, which evidences the yellowish color, is directly related to the pulps which have carotenoids in their composition (Matietto et al., 2010), as observed by the murici and tapereba pulps.

### 3.2 Total phenolic content and antioxidant activity

**Figure 1:**
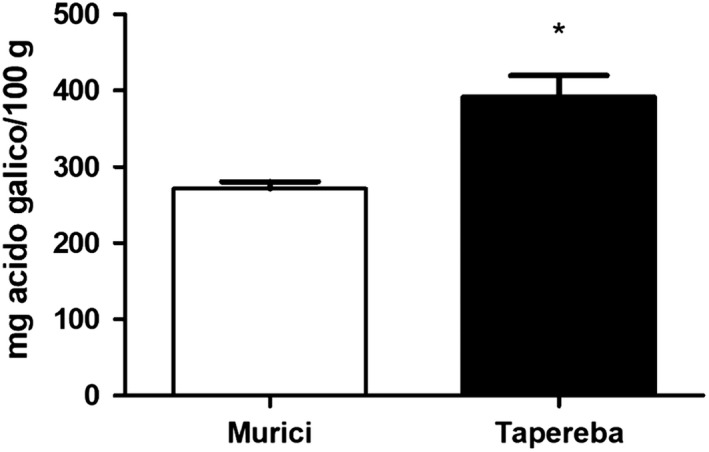
Total phenolic content (mg gallic acid (GAE)/ml) in murici and tapereba pulps. GAE = gallic acid

The content of phenolic compounds in tapereba pulp (391.69 ± 48.71 mg gallic acid/100 g) was higher when compared to murici pulp (271.60 ± 15.55 mg gallic acid/100 g) (*p* < .05) (Figure 1). In this study, we found that the total phenolic values of murici pulp were higher than those found by murici pulp in the study of Souza et al. (2012). Tapereba pulp also reported higher total phenolic values compared to the (Tiburski et al., 2011) study, which performed similar analyses to those conducted in the present study. Yellow mombin phenolic content is only lower than acerola (*Malpighia emarginata*) (580.1 mg GAE/100 g) (Bramorski et al., 2011).

Pulp of tapereba presented higher antioxidant activity according to DPPH (55.78 ± 1.65%), TEAC (64.66 ± 2.76 μM Trolox/g), and FRAP (9.45 ± 0.76 μmol sulfate/g) when compared to the murici pulp (*p* < .05). Statistical differences were observed between the murici and tapereba pulps in almost all evaluated parameters of antioxidant activity (*p* < .05), except for the antioxidant capacity of the ORAC assay, which showed similarity between the analyzed pulps (*p* > .05) (Table 2). The value of the antioxidant activity of tapereba pulp is similar to that found by Tiburski et al. (2011) according to your study with tapereba pulp. According to Souza et al. (2012), this antioxidant activity value is equivalent to some murici pulp. Antioxidant activity high in fruit pulp murici and tapereba may be associated with their vitamin C and phenolic compounds content, as well as the presence of carotenoids in their composition.

### 3.3 Experimental design

The experiments were conducted and optimized, where the pulp and sucrose were independent variables and flavor, texture, appearance, and overall acceptance were the dependent variables after the formulations and sensory evaluation of murici‐ and tapereba‐based drinks. The experiments were performed in different combinations of independent variables, and the means obtained from the analysis of acceptance are shown in Table S2.

After analyzing the acceptance test, both the murici‐based beverage formulations and the tapereba‐based beverages showed better acceptability with regard to the appearance of the attribute, which can be justified by their visually appealing yellow to orange coloration (Paakki, Aaltojärvi, Sandell, & Hopia, 2019).

**Figure 2:**
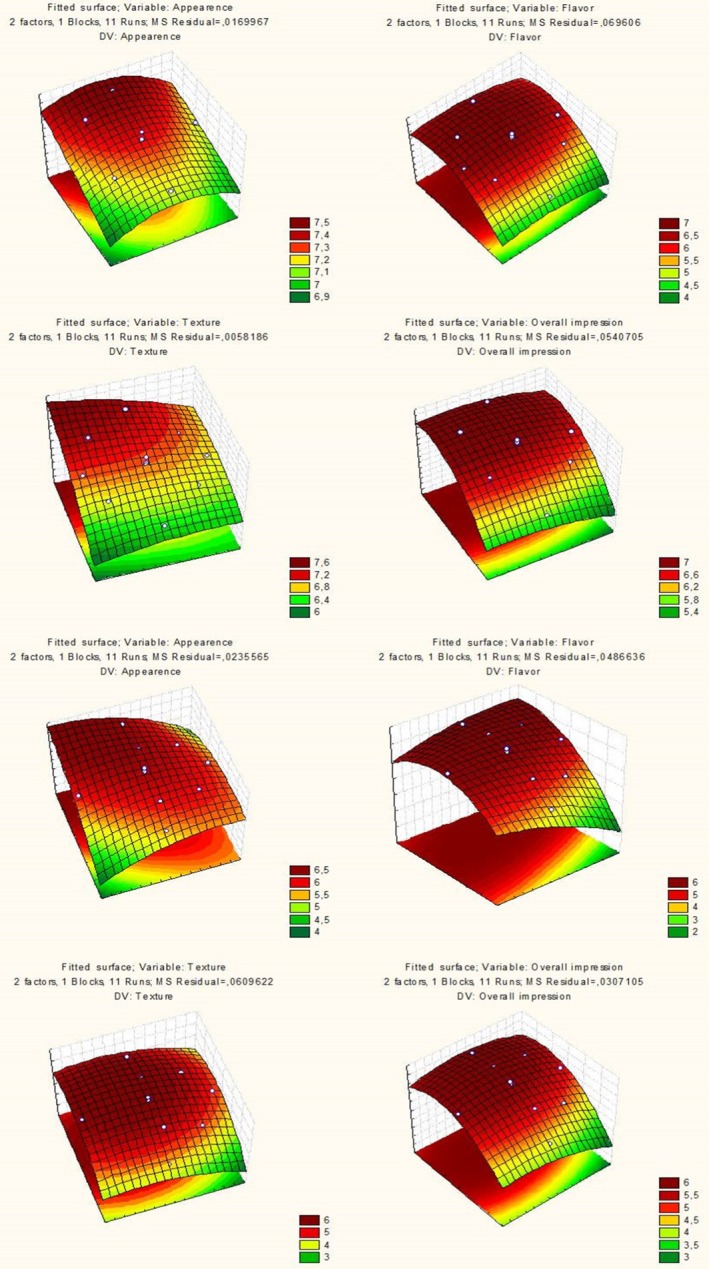
Response surface modeling three dimensions charts for the dependent (appearance, flavor, texture, and overall impression) variables of the drinks based on murici and tapereba considering the independent variables pulp and sugar

The results from the experimental data show that the pulp concentrations affected the averages of the color, texture, and overall impression attributes for the murici‐ and tapereba‐based beverage formulations (Figure 2, Figure S1). The amounts of pulp murici and tapereba fruits and sucrose used affect the acceptability of all evaluated attributes.

The influence of murici pulp on these qualities can be linked with the fact that it is a fleshy pulp with cleaner and more juicy characteristics and has the predominant bright yellow color (Morzelle et al., 2015). Tapereba pulp has influenced the acceptance of the presentation formulations with an orange and fibrous and smooth texture (Tiburski et al., 2011; Maldonado‐Astudillo 2014). These characteristics are well accepted by the tasters.

Response surface graphs, with variables that influence acceptability (Figure 2, Figure S2), show that increased sucrose has influenced the acceptance of all evaluated attributes. The fruits of the Amazon region have strong and typical characteristics of the region, the murici fruit has unique and typical flavor can be observed through the similarity with the cheese flavor, the tapereba fruit presents bitter‐sweet flavor, and adding sucrose can mask these characteristics, improving their acceptance.

Fruit‐based beverages murici and tapereba presented the best evaluations for the evaluated attributes (appearance, flavor, texture, and overall impression) in formulations 3, 7, and 11 (Table S2). Some samples were selected from this sensory evaluation and physicochemical analysis and antioxidant activity were performed. No similar formulations to those developed in the present study have been found in the literature with the fruits murici and tapereba from the Amazon region.

The formulations 7 and 11 fruit‐based murici present higher titratable acidity (*p* < .05). Such formulations had a higher amount of sugar reduction, which could be explained by the amount of pulp contained in the formulations in question, leading to a higher amount of fructose in the beverage. Formulation number 11 also revealed higher quantities of soluble solids, explained by the higher quantities of sugar used in the formulation relative to formulations number 3 and 7 (*p* < .05) (Table S3).

The amount of total phenolics in the fruit formulations 3, 7, and 11 was determined, and it was found that formulation 7 using murici pulp and formulation 11 using tapereba pulp had the highest values of total phenolic compounds.

In terms of antioxidant activity in the formulations of drinks analyzed on the basis of murici pulp 3, 7, and 11, formulation 7 in the three tests showed higher antioxidant activity compared to the others (*p* < .05), although formula 3 had the highest antioxidant activity in tapereba pulp beverage (*p* < .05).

Data analysis was used to determine the optimal formulations of fruit‐based beverages murici and tapereba, where the ideal values of each independent variable for fruit‐based beverage murici were as follows: 40% fruit pulp, 15% sugar and 45% water, and 32.5% pulp, 12% sugar, and 55% water for tapereba.

## 4 CONCLUSION

Murici and tapereba beverages are a well‐accepted product with significant nutritional features. The fruits studied are sources of bioactive compounds that provide protection for the body, and this function has been observed in the high antioxidant activity reported in the formulations, indicating that these products may bring benefits to the consumer's health. Further studies should be carried out to analyze the best production techniques, guaranteeing the quality of the beverages.

## CONFLICT OF INTEREST

The authors declare no conflict of interest.

## ETHICAL STATEMENT

The study's protocols and procedures were ethically reviewed and approved by a recognized ethical body (Research Ethics Committee of the University of the State of Rio de Janeiro—CAAE no. 39693914.8.0000.5285).

## Supporting information

Fig S1

Fig S2

Table S1

Table S2

Table S3

Suppinfo
