# Supplementary figures and images for: Fruit‐based drink sensory, physicochemical, and antioxidant properties in the Amazon region: Murici (Byrsonima crassifolia (L.) Kunth and verbascifolia (L.) DC) and tapereba (Spondia mombin)
Source: Food Sci Nutr. 2020 Apr 15;8(5):2341–7. doi: 10.1002/fsn3.1520 (PMC7215202; doi:10.1002/fsn3.1520)

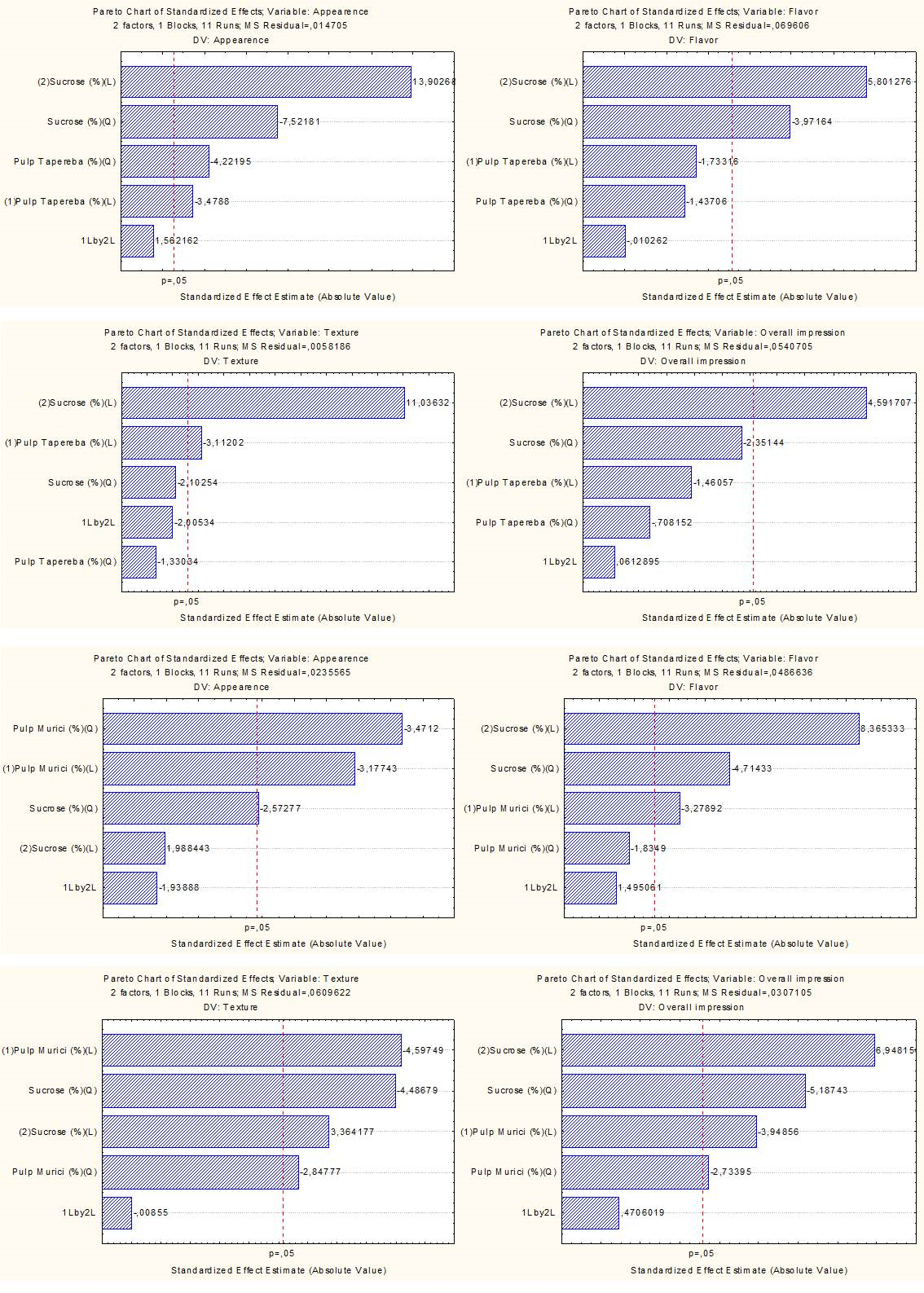

Supplement: Supplementary file 1 — Fig S1 [file FSN3-8-2341-s001.tif]

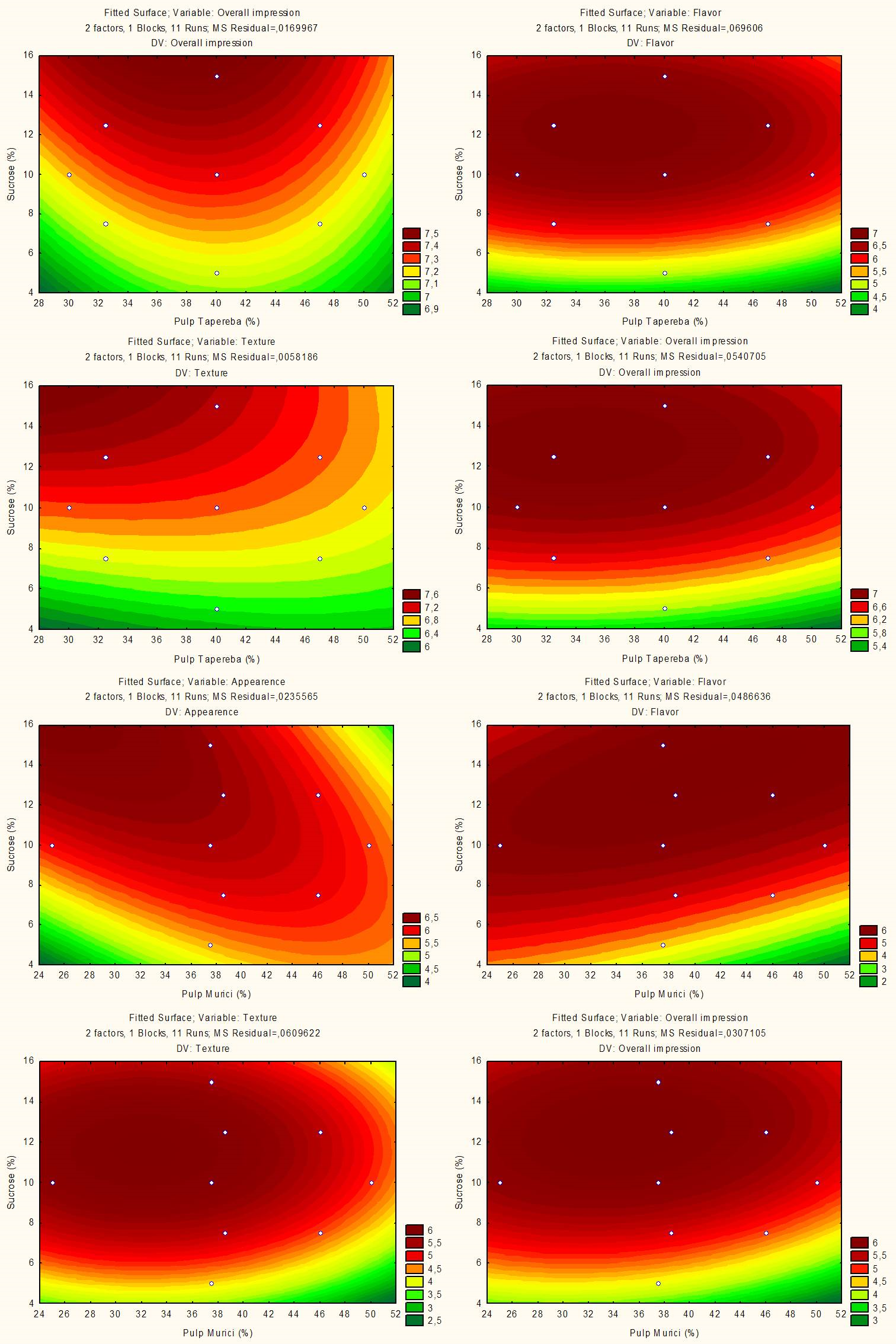

Supplement: Supplementary file 2 — Fig S2 [file FSN3-8-2341-s002.tif]
