# Supplementary material for: Fruit‐based drink sensory, physicochemical, and antioxidant properties in the Amazon region: Murici (Byrsonima crassifolia (L.) Kunth and verbascifolia (L.) DC) and tapereba (Spondia mombin)
Source: Food Sci Nutr. 2020 Apr 15;8(5):2341–7. doi: 10.1002/fsn3.1520 (PMC7215202; doi:10.1002/fsn3.1520)
Supplement: Supplementary file 3 — Table S1 [file FSN3-8-2341-s003.doc]

**Table S1.** Distribution of independent variables and respective percentage in the formulations of the fruit-base beverage murici and tapereba

| Formulations |  |  | Murici | | | | Tapereba | | |
| --- | --- | --- | --- | --- | --- | --- | --- | --- | --- |
|  | X^1^ | X^2^ | Pulp (%) | Sucrose (%) | Water (%) | | Pulp (%) | Sucrose (%) | Water (%) |
| 1 | -1 | -1 | 32.5 | 7.5 | | 60.0 | 38.5 | 7.5 | 54.0 |
| 2 | 1 | -1 | 47.0 | 7.5 | | 45.5 | 46.0 | 7.5 | 46.5 |
| 3 | -1 | 1 | 32.5 | 12.5 | | 55.0 | 38.5 | 12.5 | 49.0 |
| 4 | 1 | 1 | 47.0 | 12.5 | | 40.5 | 46.0 | 12.5 | 41.5 |
| 5 | 0 | 0 | 40.0 | 10.0 | | 50.0 | 37.5 | 10.0 | 52.5 |
| 6 | 0 | 0 | 40.0 | 10.0 | | 50.0 | 37.5 | 10.0 | 52.5 |
| 7 | 0 | 0 | 40.0 | 10.0 | | 50.0 | 37.5 | 10.0 | 52.5 |
| 8 | -1.42 | 0 | 30.0 | 10.0 | | 60.0 | 25.0 | 10.0 | 65.0 |
| 9 | 1.42 | 0 | 50.0 | 10.0 | | 40.0 | 50.0 | 10.0 | 40.0 |
| 10 | 0 | -1.42 | 40.0 | 5.0 | | 55.0 | 37.5 | 5.0 | 57.5 |
| 11 | 0 | 1.42 | 40.0 | 15.0 | | 45.0 | 37.5 | 15.0 | 47.5 |
