# Supplementary material for: Fruit‐based drink sensory, physicochemical, and antioxidant properties in the Amazon region: Murici (Byrsonima crassifolia (L.) Kunth and verbascifolia (L.) DC) and tapereba (Spondia mombin)
Source: Food Sci Nutr. 2020 Apr 15;8(5):2341–7. doi: 10.1002/fsn3.1520 (PMC7215202; doi:10.1002/fsn3.1520)
Supplement: Supplementary file 4 — Table S2 [file FSN3-8-2341-s004.doc]

**Table S2.** Distribution of the independent variables X^1^ pulps of murici and tapereba, X^2^ sucrose stipulated in the factorial planning and means obtained from the analysis of acceptance by the dependent variables Y^1^ appearance, Y^2^ flavor, Y^3^ texture and Y^4^ overall impression

|  | Murici | | | | Tapereba | | | |
| --- | --- | --- | --- | --- | --- | --- | --- | --- |
| Formulations | Y^1^ | Y^2^ | Y^3^ | Y^4^ | Y^1^ | Y^2^ | Y^3^ | Y^4^ |
| 1 | 5.83^a^ | 4.90^b^ | 5.29^c^ | 5.25^c^ | 7.25^a^ | 6.42^b^ | 6.67^c^ | 6.86^d^ |
| 2 | 5.90^a^ | 4.32^b^ | 4.43^b^ | 4.70^c^ | 7.07^a^ | 5.97^b^ | 6.60^c^ | 6.43^c^ |
| 3 | 6.22^a^ | 6.14ª^,b^ | 5.57^c^ | 5.94^b^ | 7.49^a^ | 6.98^b^ | 7.29^c^ | 7.18^c^ |
| 4 | 5.90^a^ | 5.97^a^ | 5.10^b^ | 5.71^c^ | 7.26^a^ | 6.49^b^ | 6.91^c^ | 6.75^d^ |
| 5 | 6.39^a^ | 6.18^b^ | 5.95^c^ | 6.04^b,c^ | 7.29^a^ | 6.60^b^ | 6.90^c^ | 6.78^b,c^ |
| 6 | 6.29^a^ | 5.89^b^ | 5.77^b^ | 5.88^b^ | 7.32^a^ | 6.70^b^ | 6.85^b,c^ | 6.93^c^ |
| 7 | 6.47^a^ | 6.08^b^ | 6.03^b^ | 6.06^b^ | 7.41^a^ | 6.92^b^ | 7.05^b^ | 7.05^b^ |
| 8 | 5.90^a^ | 5.89^a^ | 5.68^b^ | 5.75^b^ | 7.06^a^ | 6.46^b^ | 6.91^a^ | 6.75^c^ |
| 9 | 5.83^a^ | 5.46^b^ | 4.98^c^ | 5.41^b^ | 7.29^a^ | 6.19^b^ | 6.75^c^ | 6.68^c^ |
| 10 | 5.52^a^ | 3.95^b^ | 4.19^c^ | 4.26^c^ | 7.15^a^ | 4.71^b^ | 6.29^c^ | 5.64^d^ |
| 11 | 6.46^a^ | 6.22^b^ | 5.62^c^ | 6.06^d^ | 7.48^a^ | 6.82^b^ | 7.27^c^ | 7.17^c^ |
