# Supplementary material for: Fruit‐based drink sensory, physicochemical, and antioxidant properties in the Amazon region: Murici (Byrsonima crassifolia (L.) Kunth and verbascifolia (L.) DC) and tapereba (Spondia mombin)
Source: Food Sci Nutr. 2020 Apr 15;8(5):2341–7. doi: 10.1002/fsn3.1520 (PMC7215202; doi:10.1002/fsn3.1520)
Supplement: Supplementary file 5 — Table S3 [file FSN3-8-2341-s005.doc]

**Table S3.** Characterization of the formulations of fruit-bases beverage murici and tapereba

| **Parameters** |  | **Murici** |  |  | **Tapereba** |  |
| --- | --- | --- | --- | --- | --- | --- |
|  | **I** | **II** | **III** | **I** | **II** | **III** |
| Acidity (g%) | 0.86±0.15^a^ | 1.10±0.03^b^ | 1.16±0.05^b^ | 1.63±0.07^a^ | 2.08±0.14^b^ | 2.34±0.10^b^ |
| Reducing Sugar (g%) | 5.85±0.25^a^ | 4.32±0.19^b^ | 4.14±0.07^b^ | 10.60±4.50^a^ | 12.46±3.70^a,b^ | 17.18±2.98^b^ |
| Soluble Solids (ºBrix) | 13.00±0.01^a^ | 11.00±0.03^b^ | 16.00±0.02^c^ | 14.00±0.01^a^ | 13.00±0.01^b^ | 17.00±0.02^c^ |
| Total fenolic  (mgGAE /mL) | 12.37±0.53^a^ | 15.49±0.61^b^ | 14.33±0.41^b^ | 19.99±0.37^a^ | 22.21±0.08^b^ | 24.01±0.19^c^ |
| DPPH  (%) | 40.44±0.09^a^ | 57.53±0.11^b^ | 61.73±0.05^c^ | 37.90±0.02^a^ | 32.67±0.06^b^ | 29.44±0.10^c^ |
| TEAC  (µM Trolox/g) | 12.22±0.31^a^ | 13.07±0.12^b^ | 11.90±1.56^a^ | 18.03±0.24^a^ | 13.05±0.09^b^ | 16.32±0.27^b^ |
| ORAC  (mmol Trolox/g) | 112.60±55.30^a^ | 111.09±26.27^a^ | 104.31±34.08^b^ | 101.31±44.98^a^ | 96.51±51.02^b^ | 102.30±31.72^a^ |

Results expressed as mean ± standard deviation. Different letters on the same line indicate significant difference (p< 0.05). GAE= gallic acid.

I: Formulation 3, II: Formulation 7, III: Formulation 11.
