# Supplementary material for: Fruit‐based drink sensory, physicochemical, and antioxidant properties in the Amazon region: Murici (Byrsonima crassifolia (L.) Kunth and verbascifolia (L.) DC) and tapereba (Spondia mombin)
Source: Food Sci Nutr. 2020 Apr 15;8(5):2341–7. doi: 10.1002/fsn3.1520 (PMC7215202; doi:10.1002/fsn3.1520)
Supplement: Supplementary file 6 — Suppinfo [file FSN3-8-2341-s006.doc]

**Fruit-based drink sensory, physicochemical and antioxidant properties in the Amazon region: murici (*Byrsonima crassifolia (L*.) *Kunth* and *verbascifolia (L.) DC)* and tapereba (*Spondia mombin*)**

Vanessa Rosse de Souza^1^; Adriana Aniceto^1^; Joel Pimentel Abreu^1^; Julia Montenegro^1^; Bruno Boquimpani^1^; Vanessa Azevedo de Jesuz¹; Monique de Barros Elias Campos¹; Paulo Sérgio Marcellini^2^; Otniel Freitas-Silva^3^; Rafael Cadena^4^ and Anderson Junger Teodoro^1*^ ([atteodoro@gmail.com](mailto:atteodoro@gmail.com))

**Highlights**

- Murici and tapereba are good source of antioxidants compounds.
- Tapereba presented higher antioxidant activity compared to murici.
- Murici and tapereba beverages are a well-accepted product with significant nutritional features.

**
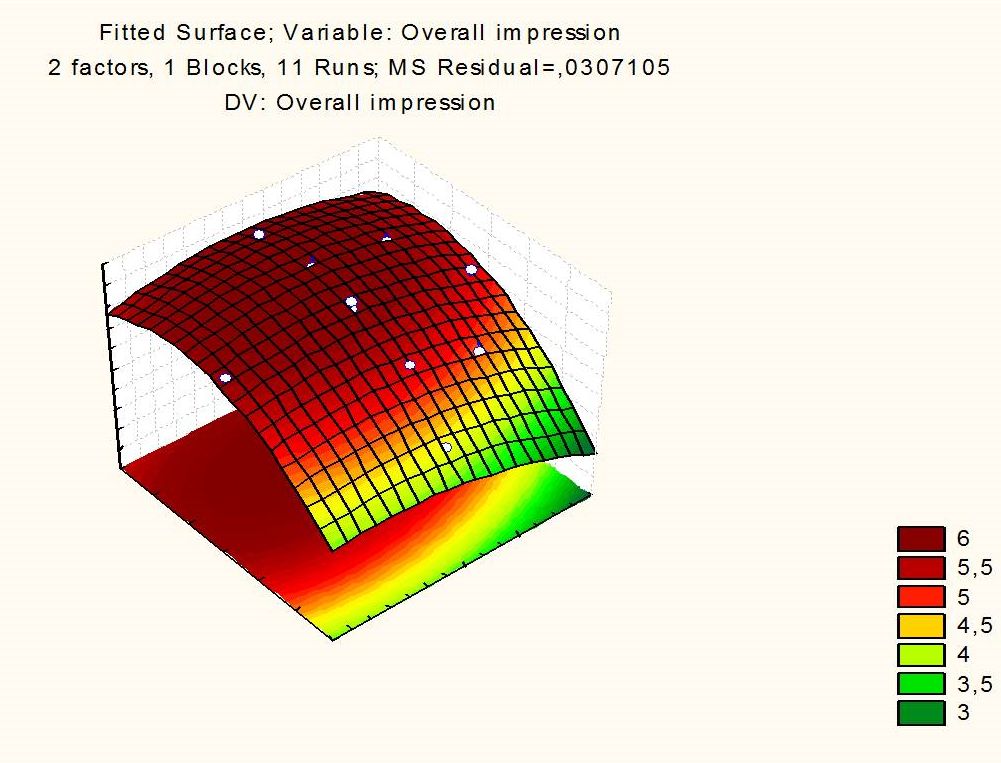
**
